# Supplementary material for: Development of peptides for targeting cell ablation agents concurrently to the Sertoli and Leydig cell populations of the testes: An approach to non-surgical sterilization
Source: PLoS One. 2024 Apr 4;19(4):e0292198. doi: 10.1371/journal.pone.0292198 (PMC10994420; doi:10.1371/journal.pone.0292198)
Supplement: S1 Fig — (DOCX) [file pone.0292198.s001.docx]

**Supporting Information**

**S1 Fig.** **Quantitative PCR (qPCR) primers:**

LHr primers

Mus musculus luteinizing hormone/choriogonadotropin receptor (Lhcgr), transcript variant 1, mRNA; NCBI Reference Sequence: NM_013582.3 and Mus musculus luteinizing hormone/choriogonadotropin receptor (Lhcgr), transcript variant 3, mRNA; NCBI Reference Sequence: NM_001364898.1

Forward: 5’–CTGGTGCTGAAGCAGTCACA-3’

Reverse: 5’-TAGGTGAGAGATAGTCGGGCG-3’)

T_M_: 61 ˚C

Product length: 137

Housekeeping gene, GAPDH, primers

Mus musculus glyceraldehyde-3-phosphate dehydrogenase Gene ID 14433 glyceraldehyde-3-phosphate dehydrogenase isoform 1 NM_001289726.2: glyceraldehyde-3-phosphate dehydrogenase isoform 2 NM_008084.4: and glyceraldehyde-3-phosphate dehydrogenase isoform 3 NM_001411841.1

Forward: 5’-GTGATGGGTGTGAACCACGA-3’

Reverse: 5’- GGTCATGAGCCCTTCCACAA-3’)

T_M_: 58 ˚C

Product Length: 135
