# Supplementary material for: Development of peptides for targeting cell ablation agents concurrently to the Sertoli and Leydig cell populations of the testes: An approach to non-surgical sterilization
Source: PLoS One. 2024 Apr 4;19(4):e0292198. doi: 10.1371/journal.pone.0292198 (PMC10994420; doi:10.1371/journal.pone.0292198)
Supplement: S2 Fig — A. Relative expression of LHr mRNA in mouse Leydig cell lines, MLTC1 and TM3 cells, whole testis and mEcap18 cells assessed by qPCR. B. Immunocytochemistry using an anti-LHr antibody with an AlexFluor 488 secondary indicates that the LH receptor is expressed at high levels by MLTC1 cells but negligibly in mEcap18 cells. (DOCX) [file pone.0292198.s002.docx]

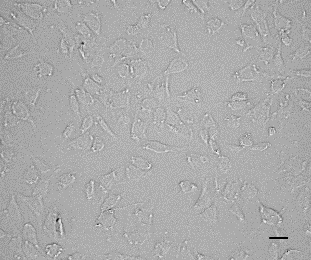

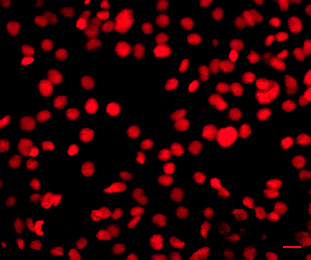

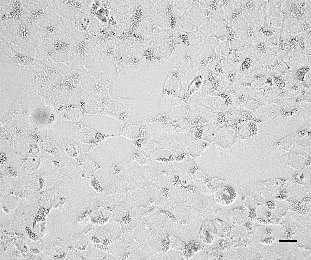


Phase

Far red nuclear stain

MLTC1 Leydig cells

mEcap18 cells


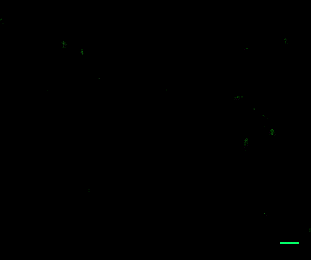

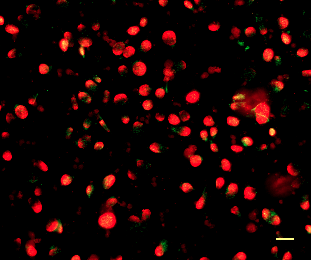

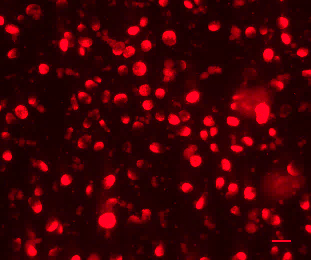

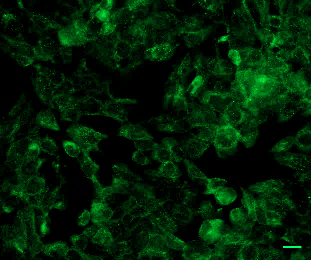

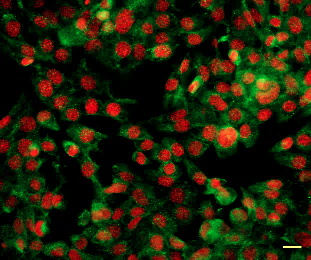


LH receptor (green)

Overlay


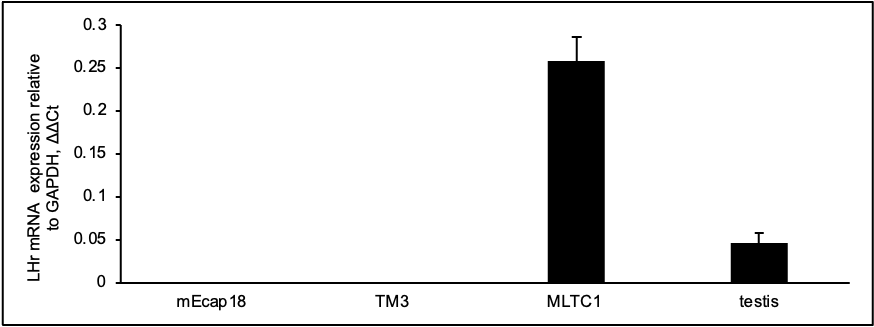


**A**

**B**

**S2 Fig**. **Choice of Leydig cell model.** **A.** Relative expression of LHr mRNA in mouse Leydig cell lines, MLTC1 and TM3 cells, whole testis and mEcap18 cells assessed by qPCR. **B.** Immunocytochemistry using an anti-LHr antibody with an AlexFluor 488 secondary indicates that the LH receptor is expressed at high levels by MLTC1 cells but negligibly in mEcap18 cells.

**S2 Fig**. **Choice of Leydig cell model.** **A.** Relative expression of LHr mRNA in mouse Leydig cell lines, MLTC1 and TM3 cells, whole testis and mEcap18 cells assessed by qPCR. **B.** Immunocytochemistry using an anti-LHr antibody with an AlexFluor 488 secondary indicates that the LH receptor is expressed at high levels by MLTC1 cells but negligibly in mEcap18 cells.
