# Supplementary material for: Development of peptides for targeting cell ablation agents concurrently to the Sertoli and Leydig cell populations of the testes: An approach to non-surgical sterilization
Source: PLoS One. 2024 Apr 4;19(4):e0292198. doi: 10.1371/journal.pone.0292198 (PMC10994420; doi:10.1371/journal.pone.0292198)
Supplement: S3 Fig — Male adult mice were injected IP with 300 μl of either 1mM peptide-FITC in 30% Kolliphor/PBS or 30% Kolliphor/PBS with 3 mice in each treatment group. Animals were euthanized 8 hours post-injection and all tissues were imaged using Leica MZFLIII microscope with an epifluorescent green filter next to the vehicle control. Peptide-FITC applied is indicated in the first image of each row. Fourth tissue in the bottom three images were an unrelated peptide-FITC. (DOCX) [file pone.0292198.s003.docx]

**Liver**

**Kidney**

**Spleen**

**Bright field**

**FITC**

**Bright field**

**FITC**

**Bright field**

**FITC**


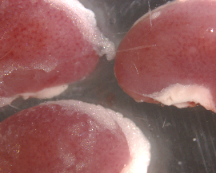

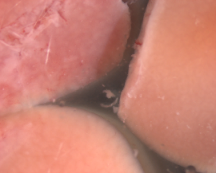

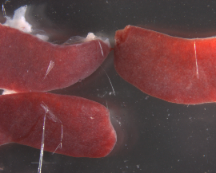

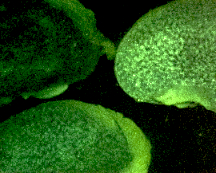

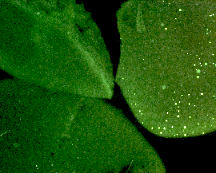

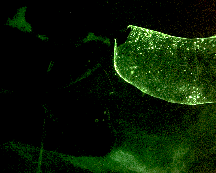


**L57-FITC**

**Vehicle**

**L101-FITC**


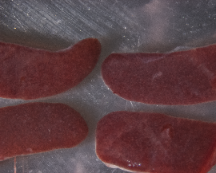

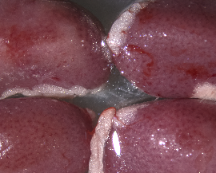

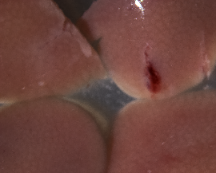

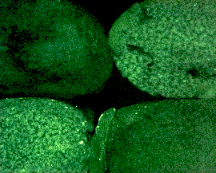

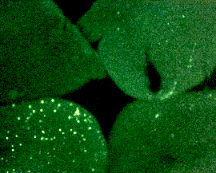

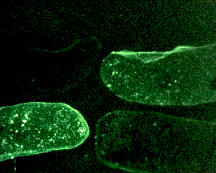


**Vehicle**

**LHa-FITC**

**L95-FITC**

**S3 Fig. Representative images of excretory organs (kidney, liver and spleen) 8 hours post-injection**. Male adult mice were injected IP with 300 µl of either 1mM peptide-FITC in 30% Kolliphor/PBS or 30% Kolliphor/PBS with 3 mice in each treatment group. Animals were euthanased 8 hours post-injection and all tissues were imaged using Leica MZFLIII microscope with an epifluorescent green filter next to the vehicle control. Peptide-FITC applied is indicated in the first image of each row. Fourth tissue in the bottom three images were an unrelated peptide-FITC.
