# Supplementary material for: Development of peptides for targeting cell ablation agents concurrently to the Sertoli and Leydig cell populations of the testes: An approach to non-surgical sterilization
Source: PLoS One. 2024 Apr 4;19(4):e0292198. doi: 10.1371/journal.pone.0292198 (PMC10994420; doi:10.1371/journal.pone.0292198)
Supplement: S4 Fig — The linked maleimide allows conjugation of the peptide via the N-terminal thiol group. Between the maleimide and the Auristatin there is a cleavable linker made up of valine, citrulline (circled in red) and para-aminobenzyl carbamate (PABC). In the lysosome, the dipeptide linker is cleaved and then Auristatin is released from PABC by 1,6-elimination. Structures drawn using software: Advanced Chemistry Development Inc. ACD/3D Viewer (Freeware) Product Version 12.01 (Build 32890, 18 May 2009). (DOCX) [file pone.0292198.s004.docx]

Lysosomal processing

+

+

+

1,6-elimination

Peptide-vcMMAE

MMAE

**S4 Fig**. **Lysosomal processing of vcMonomethylAuristatin E (vcMMAE)**, an anticancer agent that interacts with α-tubulin in a similar way to Vinca alkaloids to block α-tubulin polymerisation and prevents the formation of the mitotic apparatus. The linked maleimide allows conjugation of the peptide via the N-terminal thiol group. Between the maleimide and the Auristatin there is a cleavable linker made up of valine, citrulline (circled in red) and para-aminobenzyl carbamate (PABC). In the lysosome, the dipeptide linker is cleaved and then Auristatin is released from PABC by 1,6-elimination. Structures drawn using software: Advanced Chemistry Development Inc. ACD/3D Viewer (Freeware) Product Version 12.01 (Build 32890, 18 May 2009).
