# Supplementary material for: Development of peptides for targeting cell ablation agents concurrently to the Sertoli and Leydig cell populations of the testes: An approach to non-surgical sterilization
Source: PLoS One. 2024 Apr 4;19(4):e0292198. doi: 10.1371/journal.pone.0292198 (PMC10994420; doi:10.1371/journal.pone.0292198)
Supplement: S5 Fig — Male adult mice were injected IP with 300 μl/30 g of either 14.5 mM FSH2Menadione; 100 μl/30 g 420 μM LH2Auristatin; a combination of both, 16 hours apart; or 300 μl/30 g of the vehicle (30% Kolliphor/PBS) with 10 mice in each treatment group. Five males in each treatment group were mated, with two control females each, six weeks post-injection for three weeks. Males were euthanized immediately after the mating period (~10 weeks post-injection) A. Anti-Sox9 was used to stain for Sertoli cells in testis sections from each of five males in each treatment group. Images of the testes of the treated males. Inset is secondary only control. Scale bar = 50 μm. B. Sertoli cells were counted in all tubules of each section for 5 individuals from each treatment (n = 5). NB. Subjects were randomly selected from the treatment groups but excluded those testes weighing less than 60 mg. C. Serum FSH levels were measured using an anti-FSH ELISA (n = 9). (DOCX) [file pone.0292198.s005.docx]

**S5 Fig. Effect of FSH2Md and LH2Auristatin *in vivo.*** Male adult mice were injected IP with 300 µl/30 g of either 14.5 mM FSH2Menadione; 100 µl/30 g 420 µM LH2Auristatin; a combination of both, 16 hours apart; or 300 µl/30 g of the vehicle (30% Kolliphor/PBS) with 10 mice in each treatment group. Five males in each treatment group were mated, with two control females each, six weeks post-injection for three weeks. Males were euthanased immediately after the mating period (~10 weeks post-injection) **A*.*** Anti-Sox9 was used to stain for Sertoli cells in testis sections from each of five males in each treatment group. Images of the testes of the treated males. Inset is secondary only control. Scale bar=50 µm. **B.** Sertoli cells were counted in all tubules of each section for 5 individuals from each treatment (n=5) . NB. Subjects were randomly selected from the treatment groups but excluded those testes weighing less than 60 mg. **C.** Serum FSH levels were measured using an anti-FSH ELISA (n=9).

**A**

**B**

**C**


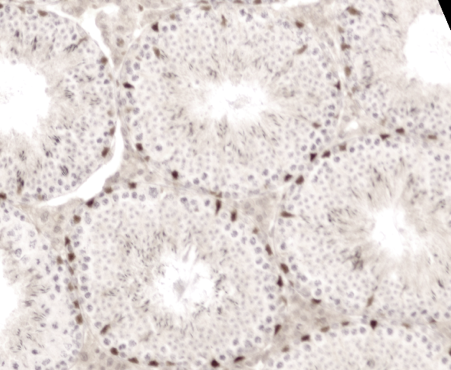

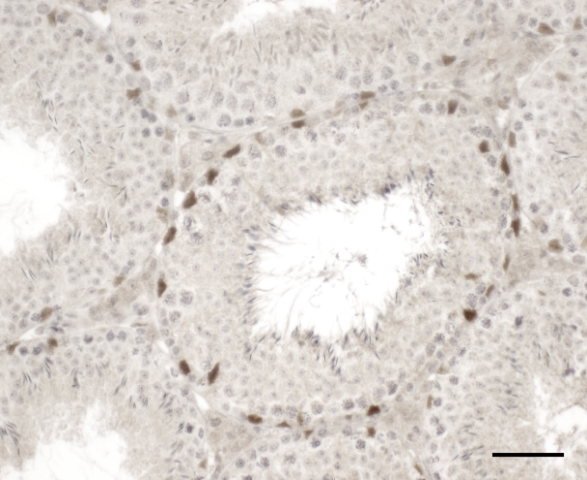

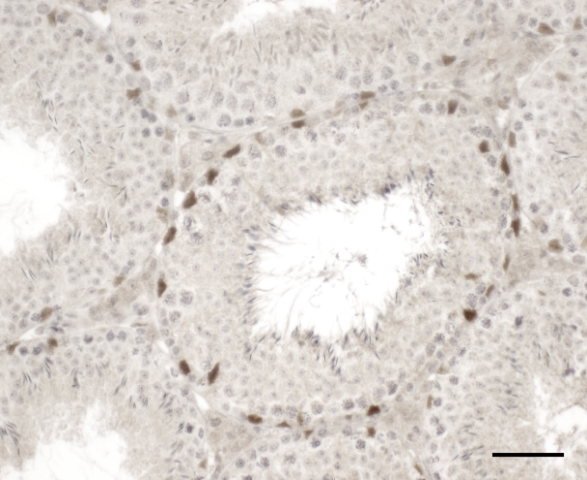

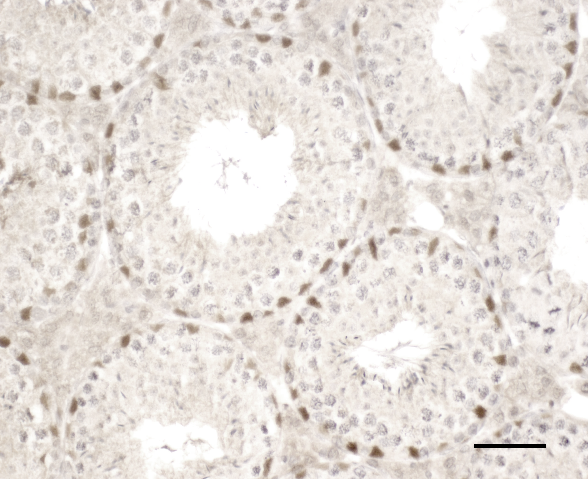

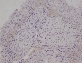


**FSH2MdLH2Aur**

**LH2Aur**

**FSH2Md**

**Vehicle**

**S5 Fig. Effect of FSH2Md and LH2Auristatin *in vivo.*** Male adult mice were injected IP with 300 µl/30 g of either 14.5 mM FSH2Menadione; 100 µl/30 g 420 µM LH2Auristatin; a combination of both, 16 hours apart; or 300 µl/30 g of the vehicle (30% Kolliphor/PBS) with 10 mice in each treatment group. Five males in each treatment group were mated, with two control females each, six weeks post-injection for three weeks. Males were euthanased immediately after the mating period (~10 weeks post-injection) **A*.*** Anti-Sox9 was used to stain for Sertoli cells in testis sections from each of five males in each treatment group. Images of the testes of the treated males. Inset is secondary only control. Scale bar=50 µm. **B.** Sertoli cells were counted in all tubules of each section for 5 individuals from each treatment (n=5) . NB. Subjects were randomly selected from the treatment groups but excluded those testes weighing less than 60 mg. **C.** Serum FSH levels were measured using an anti-FSH ELISA (n=9).
