# Supplementary material for: Development of peptides for targeting cell ablation agents concurrently to the Sertoli and Leydig cell populations of the testes: An approach to non-surgical sterilization
Source: PLoS One. 2024 Apr 4;19(4):e0292198. doi: 10.1371/journal.pone.0292198 (PMC10994420; doi:10.1371/journal.pone.0292198)
Supplement: S6 Fig — Male adult mice were injected IP with 300 μl/30 g of either 14.5 mM FSH2Menadione; 100 μl/30 g 420 μM LH2Auristatin; a combination of both, 16 hours apart; or 300 μl/30 g of the vehicle (30% Kolliphor/PBS) with 10 mice in each treatment group. Males were euthanized 10 weeks post-injection and epididymal sperm was collected from all ten males in each treatment group and all samples were assessed for vitality, motility, mitochondrial ROS, and cytoplasmic ROS. Flow cytometric analysis was used with probes to assess A. Mitochondrial ROS (MSR) and B. Cytoplasmic superoxide (DHE). C. The eosin exclusion method was used to assess the caudal sperm vitality. D. The sperm chromatin structure assay (SCSA) detected susceptibility to acid-mediated DNA fragmentation expressed here as DNA fragmentation index (DFI). Statistical analysis by One-way ANOVA showed no significant differences between the treatments. (DOCX) [file pone.0292198.s006.docx]

**S6 Fig. Epididymal sperm – effect of FSH2Menadione (FSH2Md) and LH2Auristatin (LH2Aur) *in vivo.*** Male adult mice were injected IP with 300 µl/30 g of either 14.5 mM FSH2Menadione; 100 µl/30 g 420 µM LH2Auristatin; a combination of both, 16 hours apart; or 300 µl/30 g of the vehicle (30% Kolliphor/PBS) with 10 mice in each treatment group. Males were euthanized 10 weeks post-injection and epididymal sperm was collected from all ten males in each treatment group and all samples were assessed for vitality, motility, mitochondrial ROS, and cytoplasmic ROS. Flow cytometric analysis was used with probes to assess A***.*** Mitochondrial ROS (MSR) and **B.** Cytoplasmic superoxide (DHE). **C*.*** The eosin exclusion method was used to assess the caudal sperm vitality. **D.** The sperm chromatin structure assay (SCSA) detected susceptibility to acid-mediated DNA fragmentation expressed here as DNA fragmentation index (DFI). Statistical analysis by One-way ANOVA showed no significant differences between the treatments.

**C**

**D**

**B**

**A**
