# Supplementary material for: Development of peptides for targeting cell ablation agents concurrently to the Sertoli and Leydig cell populations of the testes: An approach to non-surgical sterilization
Source: PLoS One. 2024 Apr 4;19(4):e0292198. doi: 10.1371/journal.pone.0292198 (PMC10994420; doi:10.1371/journal.pone.0292198)
Supplement: S8 Fig — Male adult mice were injected IP with 300 μl/30 g of either 14.5 mM FSH2Menadione; 100 μl/30 g 420 μM LH2Auristatin; a combination of both, 16 hours apart; or 300 μl/30 g of the vehicle (30% Kolliphor/PBS) with 10 mice in each treatment group. Five males in each treatment group were mated, with two control females each, six weeks post-injection for three weeks. Males were euthanized immediately after the mating period (~10 weeks post-injection). Haematoxylin and eosin stained sections illustrating. A. Normal development of the seminiferous tubules in a vehicle control testis section and B. Abnormal development of the seminiferous epithelia in a testis section from FSH2MdLH2Aur-treated male. Scale bar = 100 μm. (DOCX) [file pone.0292198.s008.docx]

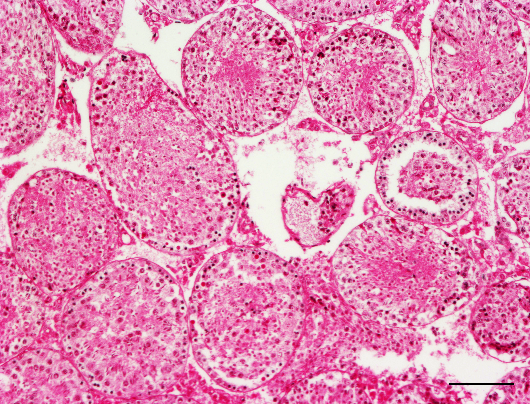

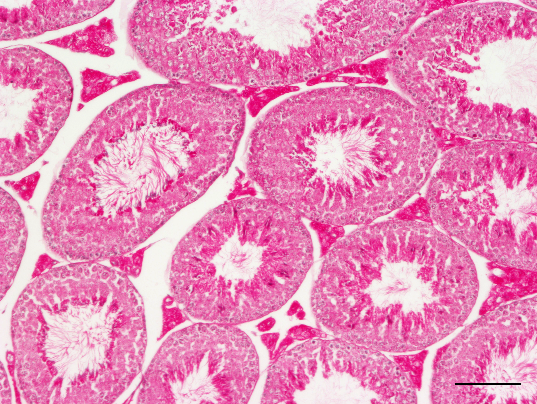


**A**

**B**

**S8 Fig. Effect of FSH2Menadione (FSH2Md) and LH2Auristatin (LH2Aur) *in vivo.*** Male adult mice were injected IP with 300 µl/30 g of either 14.5 mM FSH2Menadione; 100 µl/30 g 420 µM LH2Auristatin; a combination of both, 16 hours apart; or 300 µl/30 g of the vehicle (30% Kolliphor/PBS) with 10 mice in each treatment group. Five males in each treatment group were mated, with two control females each, six weeks post-injection for three weeks. Males were euthanized immediately after the mating period (~10 weeks post-injection). Haematoxylin and eosin stained sections illustrating**. A.** Normal development of the seminiferous tubules in a vehicle control testis section and **B.** Abnormal development of the seminiferous epithelia in a testis section from FSH2MdLH2Aur-treated male. Scale bar=100 µm.
